# Causality between various cytokines and asthma: a bidirectional two-sample Mendelian randomization analysis

**DOI:** 10.3389/fmed.2024.1447673

**Published:** 2024-08-08

**Authors:** Yansen Zheng, Qi Chen, Xiaqing Shi, Lei Lei, Donglin Wang

**Affiliations:** ^1^Medical School, Huanghe Science and Technology College, Zhengzhou, China; ^2^Jice Medical Institute, Xi’an, Shaanxi, China

**Keywords:** asthma, interleukins, interferons, tumor necrosis factors, Mendelian randomization

## Abstract

**Background:**

Many studies have shown that cytokines play an important role in the pathogenesis of asthma, but their biological effects on asthma remain unclear. The Mendelian randomization (MR) method was used to evaluate the causal relationship between various cytokines [such as interleukins (ILs), interferons (IFNs), tumor necrosis factors (TNFs), colony-stimulating factors (CSFs), transforming growth factor (TGF), etc.,] and asthma.

**Methods:**

In this study, inverse variance weighting was used to evaluate the causal relationship between asthma and cytokines. In addition, the reliability of the results is ensured by multiple methods such as MR-Egger, weighted median, MR-Raps, MR-Presso, and RadialMR, as well as sensitivity analysis.

**Results:**

The results showed that none of the 11 cytokines was associated with the risk of asthma. In contrast, asthma can increase levels of IL-5 [odds ratio (OR) = 1.112, 95% confidence interval (CI): 1.009–1.224, *P* = 0.032] and IL-9 (OR = 1.111, 95% CI: 1.013–1.219, *P* = 0.025).

**Conclusion:**

Genetically predicted asthma was positively associated with elevated levels of IL-5 and IL-9, indicating the downstream effects of IL-5 and IL-9 on asthma. Medical treatments can thus be designed to target IL-5 and IL-9 to prevent asthma exacerbations.

## 1 Introduction

Asthma is a chronic lower respiratory disease that is difficult to cure, with prevalence rates reaching 15% in children and 10% in adults, and cases continue to rise for unknown reasons (1, 2). The clinical symptoms of asthma include recurrent coughing, wheezing, chest tightness, and difficulty breathing, etc., which seriously reduces the patient’s quality of daily life and increases social medical expenses and medical burden of social services (3, 4). Therefore, there is a need for a deeper understanding of the complex molecular mechanisms of asthma to develop personalized treatments that target specific immune mechanisms.

The main features of asthma are airway inflammation and airway hyperresponsiveness. Interventional therapy targeting airway inflammation is one of the main solutions to control asthma. Several cytokines such as interleukins (ILs), interferons (IFNs), tumor necrosis factors (TNFs), colony-stimulating factors (CSFs), and transforming growth factors (TGFs) are associated with the development of airway inflammation (5, 6). Both IL-4 and IL-13 play inflammatory roles in the development of asthma and their expression is regulated. Kotsimbos et al. found that IL-13 and IL-4 were co-expressed and significantly upregulated in the bronchial mucosa of 9 asthma patients and 10 healthy controls (7). IL-4 can exacerbate asthma by inducing B-cell autophagy, promoting immunoglobulin E (IgE) production, and receptor expression (3, 7, 8). Injections of TNF-α into the airways of normal volunteers lead to increased bronchial reactivity, and prospective studies have shown that increased maternal weight leads to increased concentrations of TNF-α in the blood of infants, thereby enhancing the risk of asthma in infants (9). Sun et al. conducted a meta-analysis of 50 studies and found that TNF-α-238G/A, -308G/A, and -857C/T polymorphisms were significantly correlated with the risk of asthma in different ethnic groups (10). IFNs have immunomodulatory properties, and studies have shown that during the onset of asthma, interferon levels in the respiratory tract are significantly changed, and reduced IFN-γ production was associated with the severity of asthma (11–13).

Although studies have demonstrated a close relationship between cytokines and asthma, the causal relationship between the two has not been fully elucidated. In this paper, the causal relationship between asthma and various cytokines (interleukin, interferon, tumor necrosis factor, etc.) has been analyzed by Mendelian randomization (MR), a method of causality assessment by genetic tools, which can provide a better understanding of the mechanism of cytokine action associated with inflammation in asthma. The results lay the theoretical foundation for proposing more targeted and effective therapeutic regimens for better control of patients’ ast.

## 2 Materials and methods

### 2.1 Data sources

Genome-wide association study (GWAS) summary datasets for asthma were obtained from the IEU Open GWAS Database.^1^ We selected for analysis a variety of cytokines that are altered in the onset of asthma, and dominated by ILs, with IL-2, IL-4, IL-5, IL-9, IL-13, IL-17, IL-27, TNF-α, TNF-β, IFN-γ, G-CSF, M-CSF, and TGF-β. Among them, IL-27 and TGF-β were obtained from different GWAS of inflammatory cytokines levels (14, 15). The rest of the cytokines GWAS data were obtained from a multicenter study in Finland. Subjects aged 25–74 years were randomly selected from five regions in Finland. Cytokine data of the subjects were analyzed, and peripheral blood was extracted for expression quantitative trait loci (eQTL) analysis to quantify their gene expression profiles (16). See Supplementary Table 1 for more details.

### 2.2 Quality control and identifying genetic instruments

To confirm the independence of single nucleotide polymorphism (SNPs), we selected SNPs that exceeded the genome-wide association threshold (*p* < 5 × 10^–6^) as independent variables (IVs), because the small number of SNPs met the threshold for significance (*p* < 5 × 10^–8^); and SNPs with a physical distance less than 5000 kb and r^2^< 0.01 were removed to avoid linkage disequilibrium (LD). For SNPs not found in the outcome dataset, proxy-SNPs were used based on the LD-threshold r^2^ > 0.8, and SNPs that failed to find alternative sites were eliminated. Data were collated so that the effect and other alleles are indeed the same between exposure and outcome datasets.

### 2.3 MR analysis

This study was a bidirectional MR analysis based on a two-sample MR analysis. The inverse-variance weighted MR method (IVW-MR) was used as the main analysis to estimate the causal effects between cytokines levels and asthma. The IVW-MR method specifically requires that SNPs affect the outcome only through exposure in the study. Although many known confounding SNPs were excluded as much as possible in this study, there are still many unknown confounding factors that may lead to gene pleiotropy and bias for the estimation of effect values. Therefore, we added other methods, include MR-Egger, weighted median, MR-Raps, MR-Presso, and RadialMR, as supplements to test the reliability and stability of the results.

We performed a series of sensitivity tests to ensure that our results were robust. Cochran’s Q test was used to account for possible heterogeneity, with *p* < 0.05 deeming significantly heterogeneous, and a random-effect IVW model was applied. The horizontal pleiotropy effect was estimated by MR-Eggers. The statistical tests for MR analysis were performed using the “Two-Sample-MR” package (version 0.5.4) in the R environment (R version 4.2). The statistically significant association is defined as *p* < 0.05.

## 3 Results

### 3.1 Influence of cytokines on asthma

**FIGURE 1:**
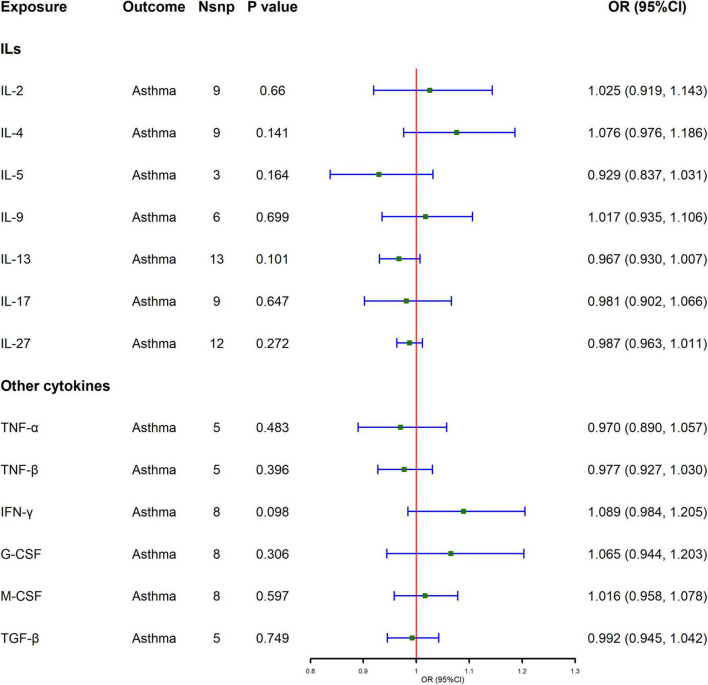
Causal effect of cytokines levels on asthma using Mendelian randomization. Odds ratio (OR) and 95% confidence interval (CI) represent the change in the odds ratio of asthma per 1-SD increase in cytokine level.

All selected cytokines were analyzed using IVW as the main analytical method, and the results are shown in Figure 1 and Supplementary Table 2. Results of the MR analysis showed that genetically determined higher levels of cytokines did not affect the risk of asthma, and the results of IVW were consistent with those of other analysis methods. For the MR-Raps analysis method, some cytokines do not have a *P*-value, because only five or fewer SNPs are within the screening conditions, making it impossible to perform MR-Raps analysis. In the sensitivity analysis, the MR-Egger intercept test suggested no evidence of pleiotropy (Supplementary Table 4). There was significant heterogeneity in the Cochran’s Q test of IL-2 and IL-27 (Supplementary Table 4), the random-effect model were performed for their IVW analyses, and it supports the existing results.

### 3.2 Influence of asthma on cytokines

**FIGURE 2:**
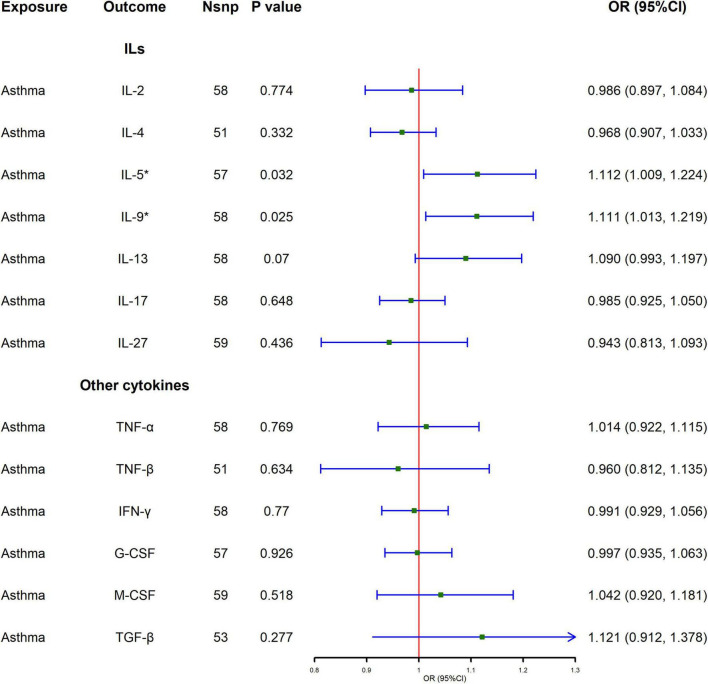
Causal effect of asthma on cytokines levels using Mendelian randomization. Odds ratio (OR) and 95% confidence interval (CI) represent the change in the odds ratio of cytokines level per 1-SD increase in the risk of asthma.

Figure 2 and Supplementary Table 3 show the MR analysis results of the impact of asthma on the levels of various cytokines. The results show that asthma is significantly correlated with elevated levels of IL-5 and IL-9 [IL-5, odds ratio (OR) = 1.112, 95% confidence interval (CI): 1.009–1.224, *P* = 0.032; IL-9, OR = 1.111, 95% CI: 1.013–1.219, *P* = 0.025], and the results of IVW are also supported by Radial-MR. In sensitivity analysis, no evidence of directional pleiotropy was found in the MR-Egger analysis. The test for heterogeneity confirmed that there is no evidence of heterogeneity (Supplementary Table 4).

## 4 Discussion

Since asthma and cytokines are closely related, we investigated the causality between asthma and cytokines through a comprehensive bidirectional MR analysis. The results of the MR analysis showed that asthma increased the levels of IL-5 and IL-9, but did not affect levels of other cytokines. There was no evidence that these cytokines increase or decrease the risk of asthma.

Several observational studies have also demonstrated changes in IL-5 levels during the onset of asthma. Compared to controls, bronchial biopsy results in both early and advanced asthma patients showed increased IL-5 mRNA expression (17, 18). Higher concentrations of IL-5 were detected in the induced sputum of patients with acute exacerbations of asthma, and IL-5 mRNA expression was associated with clinical severity of asthma (17, 19). IL-5 is produced by a variety of immune cells, such as regulating T helper 2 (Th2) cells, Class 2 innate lymphocytes (ILC2), mast cells, natural killer T (NKT) cells, and eosinophils (20). IL-5 levels increase when eosinophilic counts also increase during asthma development. The results of our MR analysis do not support the genetic prediction that IL-5 increases the risk of asthma. The discrepancy may be due to the effect of IL-5 on asthma risk through a complex immune cascade. IL-5 is considered to be the main regulator of eosinophilic increase and activation. IL-5 specifically binds to the receptor subunit IL-5Rα to regulate the differentiation, proliferation, migration, and activation of eosinophils. Upon activation, eosinophils release cytotoxins that induce functional damage to surrounding cells and tissues (21, 22). IL-5 has been identified as a therapeutic target for eosinophilic diseases and several monoclonal antibodies have been approved for clinical use in the treatment of severe asthma, for example, mepolizumab and reslizumab targeting IL-5, and benralizumab targeting the IL-5 receptor (23, 24).

The results of the MR analysis did not support that increased IL-9 levels increased asthma risk. A case-control study of 70 asthmatic patients and 77 healthy control adults aged 18–60 years. The patients’ asthma and its severity were confirmed by medical diagnosis and the pulmonary function test. Serum levels of IL-9 and the SNP of the IL-9 promoter rs2069882 were detected by molecular assay. The results showed that IL-9 serum levels were not significantly associated with asthma severity or atopic type, and the SNP of the IL-9 promoter rs2069882 was not significantly associated with asthma susceptibility (25). Similarly, Waldman and Robinson (26) meta-analysis of linkage studies between asthma and the IL-9 gene showed that the IL-9 gene has little correlation with the pathogenesis of asthma (26). Consistent with MR results, another observational study noted segmental allergen excitation in patients with atopic asthma led to increased expression of IL-9 in lymphocytes in bronchoalveolar lavage fluid (27). Elevated levels of IL-9 have also been detected in asthmatic patients’ lungs, sputum, and serum (28). In addition, elevated mRNA expression levels of IL-9 and IL-9 receptor (IL-9R) were detected in patients with asthma (29). IL-9 is derived primarily from T helper cells, a subgroup of T cells that specialize in producing IL-9, called Th9 cells. IL-9 can also be produced by different immune cells such as mast cells, macrophages, eosinophils, and neutrophils (28–30). The expression of IL-9 can promote the production of IgG and IgE in B-cell lymphocytes, promote the survival and maturation of eosinophils synergistically with IL-5, and activate airway epithelial cells by stimulating the expression of various proteases, chemokines and selective mucins (31, 32).

For other cytokines, there are some differences in our results compared to previous studies showing a strong relationship with asthma. In patients with eosinophilic asthma, the level of CSF in the respiratory tract is significantly increased, causing allergenic sensitization in the lungs, promoting neutrophils proliferation in the bone marrow, and ultimately leading to asthma exacerbation (33). Prophylactic injection of IL-27 reduced the concentration of Th2 cytokines and increased the number of type 1 regulatory T cells in mice, thereby reducing the lung inflammatory environment and improving asthma (34). In this study, there was no evidence that asthma development could increase or decrease the levels of these cytokines, and whether the changes in the levels of these cytokines had an effect on the risk of asthma. This suggests that previous observational findings may be due to environmental influences, complex regulatory mechanisms in the body, or some other unknown mechanism.

In the study, we selected a variety of cytokines whose expression levels are altered in the onset of asthma, as well as data with the large sample sizes used in most of the analyses we conducted. It provides some reference for further study. Secondly, MR analysis can reduce the influence of confounding factors and reverse causality to a certain extent. Both cytokines and asthma-related genetic IVs are derived from European population studies, which can greatly reduce the bias caused by population stratification.

However, there are some limitations to this MR study. Firstly, the samples selected for this paper were patients who were clearly diagnosed with asthma after various tests in hospital visits, there was no sub-type categorization of asthma to allow for in-depth study of a specific asthma group, and the pooled data of multiple phenotypes made it possible for some small bias in the results. Secondly, regulatory mechanisms or cascading responses during growth and development may attenuate the effects of genetic tools. MR analyses may reflect the effects of exposure to elevated cytokines on asthma throughout the human lifespan. However, high levels of cytokines presented only at a certain time of life (e.g., middle age and old age) may contribute to the risk of asthma development. Finally, the study originated from a European population, which may be partially different from other populations and cannot be applied to other Asian populations, African populations, and others. The main reason for selecting a European population is that the sample sizes of relevant GWAS studies in European populations are relatively larger and the data is more comprehensive, making the results more reliable.

## 5 Conclusion

The MR analysis showed that asthma increased the levels of IL-5 and IL-9, and there was no evidence that cytokines increased or decreased the risk of asthma. Future work could further investigate whether altered levels of IL-5 and IL-9 can be used as biomarkers of asthma development, and further explore the potential mechanism of action of IL-5 and IL-9 in asthma, and evaluate their potential for clinical prevention and treatment of asthma.

## Data availability statement

The original contributions presented in this study are included in this article/Supplementary material, further inquiries can be directed to the corresponding author.

## Author contributions

YZ: Conceptualization, Project administration, Software, Writing – original draft, Writing – review and editing. QC: Conceptualization, Formal analysis, Investigation, Writing – review and editing. XS: Conceptualization, Data curation, Validation, Writing – review and editing. LL: Data curation, Validation, Visualization, Writing – review and editing. DW: Conceptualization, Resources, Writing – original draft, Writing – review and editing.

## Footnotes

1
https://gwas.mrcieu.ac.uk/
